# Informal Trade of Psychoactive Herbal Products in the City of Diadema, SP, Brazil: Quality and Potential Risks

**DOI:** 10.1155/2013/894834

**Published:** 2013-06-02

**Authors:** Julino Assunção Rodrigues Soares Neto, Edna Myiake Kato, Adriana Bugno, José Carlos F. Galduróz, Luis Carlos Marques, Thiago Macrini, Eliana Rodrigues

**Affiliations:** ^1^Universidade Federal de São Paulo, Departamento de Medicina Preventiva, Rua Borges Lagoa, 1341 2° andar, 04038-034 São Paulo, SP, Brazil; ^2^Universidade Federal de São Paulo, Departamento de Ciências Biológicas, Centro de Estudos Etnobotânicos e Etnofarmacológicos, Rua Prof. Artur Riedel 275, 09972-270 Diadema, SP, Brazil; ^3^Universidade de São Paulo, Departamento de Farmácia, Avenida Prof. Lineu Prestes 580, Cidade Universitária, 05508-000 São Paulo, SP, Brazil; ^4^Instituto Adolfo Lutz, Centro de Medicamentos, Cosméticos e Saneantes, Avenida Dr. Arnaldo 355, 01246-902 São Paulo, SP, Brazil; ^5^Universidade Federal de São Paulo, Departamento de Psicobiologia, Rua Botucatu 862, Edifício Biomédicas, 1° andar, 04023-062 São Paulo, SP, Brazil; ^6^Universidade Bandeirante Anhanguera, Mestrado Profissional em Farmácia, Rua Maria Cândida 1813, 5° andar, 02071-013 São Paulo, SP, Brazil; ^7^Universidade Federal de São Paulo, Departamento de Ciências Biológicas, Rua Arthur Riedel, 275-09972-270 Diadema, SP, Brazil

## Abstract

The present study aimed to assess the quality and risks involved in the consumption of psychoactive herbal products (PHs) that are available through informal commerce in the city of Diadema, SP, Brazil. Methods of ethnography were used to conduct the fieldwork during which four dealers were selected to record the collection, handling, packaging, types of PHs marketed, and their therapeutic purposes. In addition, lots of the PHs selected were purchased from the dealers and analyzed using microbiology and pharmacognosy techniques. 217 PHs were recorded and categorized into two main groups: *stimulants* (67%) and *depressants* (27%) of the central nervous system; sixteen of them were selected, and their 52 lots were acquired. The deficiencies observed in handling and packaging these lots by dealers were confirmed by microbiological analysis; 80.8% of them presented risk according to the indicators defined by the Brazilian Pharmacopoeia. The pharmacognostic analysis confirmed the authenticity of only 9 to 16 PHs analyzed. In addition, descriptions of contraindications, adverse reactions, and drug interactions were found in the literature for the PHs. The results of this study allow the observation of the priorities for the sanitary adequacy of the popular trade of herbs.

## 1. Introduction

There are several legal definitions for herbal products (HPs). In this study, HPs involve the following definitions: herbal drugs, which are mainly whole, fragmented, or broken plants, parts of plants, algae, fungi, or lichen, in an unprocessed state, usually in dried form but sometimes fresh; herbal teas which consist exclusively of one or more herbal drugs intended for oral aqueous preparations by means of decoction, infusion, or maceration [1]. According to Delay et al. [2], psychoactive herbs are those that act by modifying physiological and behavioral aspects of human beings, such as cognitive ability, patterns of thought, and humor. According to the physiological changes that the plant causes, the HPs may be classified as stimulants, depressants, or hallucinogens.

Although the use of medicinal plants and/or HP is part of human history, few researchers have been dedicated to understanding the complications and risks that may arise from the use of such “medicine,” including Barnes et al. [3] and Elvin-Lewis [4].

In Brazil and many other countries, there is informal trade of HPs in the streets without quality control, sanitary inspection, and scientific evidence. The most prominent problems in this market suggest the use of wrong species, substitutions (sometimes intentional), problems with labels, and microbial contamination, leading to inconsistency in quality. Naturally, the lack of quality control leads to uncertainty in the safety and efficacy of the plant's therapeutic use as a raw material, resulting in risks to consumers [5, 6].

Accordingly, this study is aimed at interdisciplinary research about the quality and potential risks involved in the consumption of psychoactive herbal products (PHs) available in the informal commerce in the city of Diadema, SP, Brazil.

## 2. Methodology

This project was approved by the Ethics Committee (CEP) of the Universidade Federal de São Paulo (CEP 1672/07). The PHs' dealers who agreed to participate in the study signed a consent form.

### 2.1. Ethnopharmacological Survey

Fieldwork was conducted by one of the authors (Soares, JAR), from November 2006 to July 2009 in Diadema, located 17 km from São Paulo. For this purpose, we used the following methods and techniques of ethnography: participant observation, informal and semistructured interviews and notes in a field diary [7, 8] allowing the selection of four respondents (PHs' dealers), and the recorded data obtained by applying two data sheets: *ethnopharmacological data *(including questions about commercialized PHs, forms of preparation, route of administration, doses, contraindications, obtaining, handling, and packaging) and *socioeconomic data* (including information on how the dealers learned details of the trade in PHs, age, place of birth, sex, education, and income). A sample of the PHs indicated was selected; lots of them were acquired from each of the interviewees and analyzed using pharmacognosy and microbiology techniques. Since not all respondents marketed all of these PHs, the number of lots of each PH analyzed varies from 1 to 4. Thus, for PHs that were marketed by all dealers, four lots were analyzed. Available in our earlier publication [9] are the criteria for the selection of these samples and details about the methodology used in this ethnopharmacological survey.

### 2.2. Analysis of Quality

#### 2.2.1. Pharmacognostic Analysis

*Morphoanatomical. *The lots were analyzed to verify their authenticity according to the pharmacopoeias or the literature. In addition, we observed whether there was contamination or adulteration by macroscopic characterization with the naked eye and stereoscopic magnifying glass. Histological sections from plant organs were prepared as previously described and documented [23].

#### 2.2.2. Chromatographic Profile

The powdered HPs in a grinder of knives and hammers were extracted with suitable solvents to the group of substances to be evaluated. Then, thin-layer chromatographic analyses, using a substance or reference sample extract, were performed [24, 25]. The disapproved lots in the morphoanatomical characters were not submitted to chromatographic analyses. Star anise, *Matricaria* flower, St. John wort, lemon balm, *Ginkgo*, ginseng, lime flower, and valerian were analyzed according to the European Pharmacopoeia [26]. While passion fruit, guarana, marapuama, clove vine, and mulungu were analyzed according to the Brazilian Pharmacopoeia [25, 27]. Finally, reference substances/extracts were used in the analyses of node of dog [28], catuaba [29–31], and Brazilian ginseng [32, 33]; these analyses were performed according to the literature.

#### 2.2.3. Microbiological Analysis

The lots were analyzed to verify their possible microbial contamination. They were evaluated for the load of bacteria and fungi present and the presence of microorganism indicators of risk for oral administration—*Salmonella* spp., *Escherichia coli*, *Pseudomonas aeruginosa*, *Bacillus cereus*, *Enterobacter* spp., *Candida albicans*, *Aspergillus flavus,* and *A. parasiticus*—as in Brazilian Pharmacopoeia [34]. In addition, we observed the potential of mycoflora isolated to produce aflatoxins, ochratoxin A, and citrinin.

Microbiological analyses were performed as described in official compendia [6, 25]. Those analyses for the enumeration of heterotrophic bacteria and fungi used the technique of sowing depth; those for the enumeration of *E. coli* and other enterobacteria used the technique of multiple pipes; and those for the isolation and identification of bacteria used selective culture media and differential staining techniques and biochemical tests.

We performed the isolation of fungi in potato dextrose agar, incubated at 26 ± 1°C for 10 days to identify the taxonomic schemes of Rapper and Fennel [35] and Pitt [36], for observation of morphological and micromorphological features.

To evaluate the toxigenic potential of *Aspergillus* and *Penicillium* isolated from the lots, we conducted inoculation in coconut agar pH 7.0 + 0.1 (to evaluate the potential to produce aflatoxins and ochratoxin A) and coco agar pH 5.0 + 0,1 (to evaluate the potential to produce citrinin), incubated at 26 ± 1°C for 10 days [37]. After incubation, the colonies were transferred to glass bottles with a large opening, which were weighed and soaked in chloroform at a rate of 3.0 mL per 1.0 g of material. The mash was kept under stirring for 30 minutes in a horizontal mechanical shaker and then filtered with filter paper. The filtrate was collected in a test tube and evaporated in a water bath at 80°C under a hood allocated to CSA. Mycotoxins were detected by thin layer chromatography, as described by Soares and Rodriguez-Amaya [38] and confirmation of the chemical identity of the mycotoxins was performed by appropriate techniques.

### 2.3. Analysis of Potential Risks

To facilitate the analysis of the potential risk of the consumption of PHs investigated in this study, Table 1 was organized containing ethnopharmacological, microbiological, and pharmacognostic data regarding the 16 PHs selected. A literature review was conducted to verify the existence of adverse reactions, drug interactions, and contraindications for these PHs. The databases consulted included the following: Micromedex, FDA Poisonous Plant Database, Scopus, SciELO, PubMed, Capes, Google Scholar, FDA Consumer, Science Direct, and SpringerLink. We used the following key words for the literature review (“Adverse reaction” OR “Adverse events” OR “Side effects” OR “Pharmacovigilance” OR “Toxicology” OR “Hospitalization” OR “Death” AND scientific name of the PH); we chose to restrict the bibliographic survey only for human trials; in the absence of data we utilized some preclinical trials to improve the discussion.

## 3. Results and Discussion

### 3.1. Socioeconomic Data

All dealers selected are men and originating from rural areas of the northeast Brazil. They declared themselves evangelical practitioners and reported that it was in their place of origin, within the family group and community, where they started learning about medicinal plants and applicants in their localities. Today many factors such as books, internet, television, and even the customers are sources of knowledge. Working time (trade PHs) showed large variations, between six and 54 years. Apparently the average customer was not proportional to the time/work experience of marketer, getting between 10 and 50 clients/day, according to what is declared. The declared gross income was between $150.00 and $2.750,00 and the net income between $58.00 and $2.125,00. All had low education and did not complete elementary school.

### 3.2. Trade of PHs in Diadema

Deficiencies were observed in the acquisition, handling, and packaging of these PHs. All dealers buy the PHs exclusively from wholesalers in the central region of São Paulo, without worrying about their authenticity. They buy and sell the PHs based on their popular names. It was observed that in most cases the dealers do not know the importance of their scientific identification. Many are purchased in powder form, which facilitates further tampering. The replacement of the stock is performed on average every two weeks. The PHs are bought in bulk and fractionated for sale at the place of business or at home. Two of the respondents, after fractioning, use a custom label on the packaging, while others simply keep the original bags in the tent open and sell drugs by spoonfuls or portions (“one hand”) packed in a paper bag. The storage of PHs is itself one of the most critical points, as dealers do not have a system that allows operation of the stock to preserve the HPs quality and validity and to avoid mixing of lots.

### 3.3. Profile of PHs

The average value of PHs was $1.50 a unit (the bag), but this can vary up to $15.00. During the interviews, 217 PHs were cataloged and classified into three groups: stimulants (67%), depressants (27%), and stimulants and depressants (6%) of central nervous system. Thus, the terms quoted by dealers are as follows: “calm,” “sedation,” “epilepsy,” “hysteria,” “anguish,” and “relaxing” which were categorized as depressants, while the uses “tonic,” “impotence,” “aphrodisiacs,” and “to improve memory” were categorized as stimulants. The more frequent preparation methods were decoction (43%) and infusion (25%). The leaves and flowers (soft tissue) were used in the form of infusion, and the hard parts such as skins, seeds, and roots were used in the form of decoction. All PHs in powder form had the indication to be solubilized in water, milk, or juice, and in some cases it was recommended to put the powder in the food. The route of administration was always oral. The dosage and administration of tea (how many times a day) varied mainly based on the age of the client and on disease severity. Additionally, the dealers showed strong resistance to prescribing teas for children and pregnant women. They prescribe half the dose for children. Babies can hardly swallow the HPs, except for matricaria flower and fennel prescribed for colic. Some studies have been carried out in Brazil with healers that sell HPs in streets and open air [39–45]. They show a great diversity in the acquisition forms and preparation of the HPs.

The Brazilian media, in general, perpetuates the popular belief that medicinal plants and their derivatives are at low risk or are exempt from risks in the treatment of diseases. With the development of pharmacovigilance, we note that in recent years it has been remarkable, especially in scientific publications, that errors in identification of plant species, fraud, tampering, contamination, heavy metals, and interactions with drugs have caused previously little known adverse events [46–49].

Sixteen of 217 PHs were selected and their 52 lots were analyzed microbiologically and pharmacognostically (Table 1). These analyses allowed us to verify the authenticity of 28 (53.5%) of 52 lots, that is, 9 PHs (star anise: 2 lots, matricaria flower: 3, clove vine: 3; *ginkgo*: 4, guarana: 4, passion fruit: 4; marapuama: 4; lime flower: 2; valerian: 2). From the 28 lots, 12 had foreign matter, and 9 had contamination by other plant organs in the upper limit permitted by the pharmacopoeias. They are matricaria flower: 1 lot; *ginkgo*: 2; guarana: 3; passion fruit: 3; three lots were found to contain presence of insects (matricaria flower: 1 lot; *ginkgo*: 1; and guarana: 1). The remaining 24 lots, regarding 7 PHs confronted the pharmacopoeial monographs or, literature by the popular name handwritten on the packaging, were disapproved by noncoincidence with the PH described (catuaba: 4 lots; St. John wort: 3; ginseng: 4; jatobà: 3; lemon balm: 2; mulungu: 4; node of dog: 4). One problem that hinders the identification of PHs, originating from various locations and even import, is the use of different vernacular names for each particular plant in different regions.

The name “catuaba” includes several plants, among which are *Anemopaegma arvense* (Vell.) Stellfeld & JF Souza (Bignoniaceae), *Erythroxylum vacciniifolium* Mart., *E. subracemosum* Turcz (Erythroxylaceae), *Tetragastris catuaba* Soares da Cunha (Burseraceae), and *Trichilia catigua* A. Juss. (Meliaceae) [50–52]. Although the Brazilian Pharmacopoeia first edition has certificated the subterranean organs of *Anemopaegma mirandum* (Cham.) Mart. ex DC. as “catuaba,” they were found in local market samples of the bark of *Trichilia catigua*, a fact previously reported by Marques [29]. The bark of their stems in combination with other plant extracts has been used in energy drinks. The species has been little studied in some animal trials, which suggest antinociceptive and antidepressant activity. The antidepressant activity was associated with modulation of the serotonergic and dopaminergic systems [13, 53, 54]. 

The mechanism of antidepressant activity of *T. catigua* has been suggested as similar to that of *Hypericum perforatum* L. (Hypericaceae), a plant known as St. John wort, which has indications for mild to moderate depression. The samples acquired in the trade did not correspond to the morphological characteristics of *H. perforatum*, which may correspond to the species *Ageratum conyzoides* L. (Asteraceae), also popularly called St. John wort in Brazil, but with anti-inflammatory activity [55]. The lots of ginseng did not coincide with the species described in pharmacopoeia (*Panax ginseng* C.A.Mey.-Araliaceae) nor with *Pfaffia paniculata* (Mart.) Kuntze-Amaranthaceae, known as “Brazilian ginseng” [33]. For the history of marketing in Brazil and chromatographic profile these samples correspond in fact to the roots of *Pfaffia glomerata* (Martius) Kuntze (Amaranthaceae), a species known as “Brazilian ginseng” or “suma” and more easily found in trade [32]. 

The “jatobà” is not registered in pharmacopoeias, but Oliveira et al. [56] describe the morphology and anatomy of its fruit as the parts used as a medicine. The lots collected consisted of stem bark, which has led to its reproof. 

The materials called “mulungu” consisted of stem bark and part of the wood, of *Erythrina* species, which are common in the country. The description present in the pharmacopoeia [27] for *E. mulungu* Mart. ex Benth. (*E. verna* Vell.) (Fabaceae) is brief, consisting only of characteristics common to the genre such as *princkly *and* ornate bark,* phloem with sclerenchyma, crystals in the phloem parenchyma, broad and conspicuous rays with starch, and fibers arranged in loose tangencial groups. Although the morphoanatomical study showed characters common to *Erythrina* species, the comparative thin layer chromatographic profile using authentic sample of *E. mulungu* and a reference compound (hesperidin) appeared distinct [24]. No similarities were found between them, suggesting that different species of the genus are marketed under the vernacular name. 

Although it was expected that the lots acquired as “node of dog” coincided with the underground organs of *Heteropterys aphrodisiaca* O. Mach. (Malpighiaceae), it was found that in the tents selected for the study this herb is replaced by the weed *Vernonia cognata* Less. (Asteraceae), known as “fish-bakes purple” and “purple cambarazinho” [28, 57]. *H. aphrodisiaca* is employed as an adaptogen, in the bottled form [58], and shows a protective effect in the germinal epithelium of the male rat reproductive system [59]. No adverse effects were observed during its consumption as infusion [60]. 

Microbiological parameters, which are considered risk indicators defined by the Brazilian Pharmacopoeia [34], showed that 42 of the 52 lots analyzed were at odds, that is, 80.8%. Although the official compendia [6, 25] recommend the absence of *Aspergillus flavus* and *A. parasiticus* in products intended for oral administration because of concern for possible contamination by aflatoxins, we verified the presence of other potentially mycotoxigenic fungal isolates among eight genera detected (*Aspergillus ochraceus, Aspergillus niger,* other *Aspergillus, Penicillium citrinum, *other* Penicillium,* and *Trichoderma*). This occurrence is in accordance with several studies on HPs [61–67]. In assessing the potential toxigenicity of the isolates of *Aspergillus* and *Penicillium *in the production of aflatoxins (B1, B2, G1, and G2), ochratoxin A and citrinin revealed that only four lots containing isolates of *A. flavus* showed the ability to produce aflatoxins, three of which demonstrated the capacity to produce only aflatoxin B1 (clove vine: 1 lot; marapuama: 1; and lime flower: 1) while one lot of St. John wort has the potential to produce aflatoxins B1 and B2. However, even though some isolated fungi presented mycotoxigenic potential, it is not possible to presuppose that there are mycotoxins in the product, since the expression of this toxicogenic potential depends on favorable conditions as regards temperature and humidity [61, 66, 67].

### 3.4. Potential Risks

For the analysis of potential risks involved in consumption of PHs in the present study, we add the data posted above resulting from our pharmacognostic and microbiological analyses and data from the literature found for the nine PHs that had their authentication confirmed in this study (Table 1). For each PH, we obtained data on adverse reactions, contraindications, or interactions, indicating a need for medical monitoring of their consumption. These analyses indicate possible risks in their consumption because the patient who seeks such PHs rarely communicates with his or her doctors about their simultaneous usage with allopathic medicines, ignoring drug interactions. Moreover, because they are sick, some patients may be immunosuppressed, so the use of drugs contaminated by certain fungi and bacteria can lead to worsening of the disease and may even favor the expression of others. Still, most of these PHs are contraindicated during pregnancy, according to the data (Table 1), which may cause risks to both pregnancy and breastfeeding. Additionally, the lack of effective regulatory actions and populational studies on their use makes it difficult to estimate the health risks associated with those products as regards the presence of microorganisms, and mycotoxins.

### 3.5. Future Aspects

 It is estimated that about 80% of the world population have already used herbal products. This is due to the increase in the popularity of the products in the United States, Europe, and other parts of the world. Moreover, they have been successfully used throughout the history of Eastern cultures. 

 However, the popular use of those plants requires some care [68, 69], especially in countries where its purchase is free. Consequently, the WHO has recently classified herbal products as a target for pharmacovigilance, encouraging member states to strengthen supervision and regulation of those products, also reinforcing the fact that it is a priority to identify the risks associated with their use. Additionally, it warns that the difficulty to access adequate information and the specific technical requirements can hinder the process [70]. Nevertheless, since supervision of those products lacks quality, they bare not considered a priority, in spite of their negative impact on public health [71]. 

In Brazil, the government is making many efforts to control the marketing of herbal medicine [72] which has received greater relevance with the publication of the Policy and the National Program on Medicinal Plants [73]. However, there is still much to be done to establish a standard of ideal quality. According to American Botanical Council [74] this problem also occurs in many other countries, but important initiatives can be taken in a joint effort between the three sectors involved (government, business, and civil society), as occurs in the USA today.

## 4. Conclusions

The results obtained by the different analysis presented here indicate the risk of consumption of the 16 PHs analyzed in light of their contamination by species of microorganism risk indicators (15 PHs) which do not match the species listed in pharmacopoeias (7 PHs) and their contamination with foreign materials (4 PHs). In addition to the problems in the present study, we also found an extensive repertoire of adverse reactions described in scientific literature for the nine PHs that had their pharmacognostic authentication confirmed by our analysis.

We conclude that the acquired PHs require improvements in the packaging, labeling, and quality suitable for the proposed purpose of local traders. These data emphasize the need for sanitary inspection and guidance to local traders to observe the quality of the HPs to be ingested by consumers. With recent economic interest in medicinal plants, it is necessary to investigate the nature of adulteration. Diversion of quality is an economic loss to the consumer. We must create a framework conducive to the necessary fitness of the industry, and we should evaluate our ability to monitor the quality of these HPs and support training programs.

Moreover, there is an urgent need to measure the impact of folk medicine with the use of PHs in cases of adverse reactions, drug interactions and worsening of disease, and the development of more scientific research to define the risks/benefits involved in their consumption.

## Figures and Tables

**Table 1:** 16 PHs investigated in this study and their ethnopharmacological, microbiological, and pharmacognostic data and published reports indicating their possible risks of use.

16 PHDs (52 lots)	Ethnopharmacology Psychoactive use (parts)	Microbiology (no. of lots containing the following microorganisms)	Pharmacognosy authenticity (no. of lots containing foreign material)	Literature data indicating risk of use
Star anise (2 lots analyzed)	Depressant and stimulant (seeds)	Not detected	(+) *Illicium verum* Hook. f. (Schisandraceae)	Toxicity: neurotoxicity has been associated with the use of star anise infusions in infants. The toxicity is attributed to adulteration or contamination of Chinese star anise (*Illicium verum*) with Japanese star anise (*I. anisatum*), which contains toxic sesquiterpene lactones such as anisatin [10]. According to Alonso [11] this plant also contains shikimin, and substances with proven cardiotoxic activity

*Matricaria* flower (3)	Depressant and stimulant (flowers)	*Enterobacter * spp., (2 lots)	(+) *Matricaria recutita* L. (Asteraceae) (2 lots)	Contraindications: hypersensitivity to matricaria flower or other members of the Asteraceae family, atopic high fever or asthma, and pregnancy. Pregnancy category: internal consumption of the whole plant should be avoided during early pregnancy. Adverse effects: anaphylaxis, emesis in high doses, conjunctivitis, eye-lid angioedema, contact dermatitis, eczema, and rhinitis. Interactions: anticoagulants [10]. There may also be interaction with potential anticoagulant plants and other herbal medicines which also alter coagulation, such as garlic, ginseng, ginger, *ginkgo*, *angelica*, anise, *arnica*, fenogreco, and willow. Interaction with other depressants of the CNS, since theoretically the concomitant use of herbal matricaria flower with other CNS depressants may increase the therapeutic and adverse effects, including valerian, kava-kava, calamus, lemongrass, among others [12]

Catuaba (4)	Stimulant (stem barks)	*Enterobacter *spp., *Aspergillus flavus* (4)	(−) *Anemopaegma mirandum* (Cham.) Mart. ex DC. (Bignoniaceae) corresponds to the stem barks of *Trichilia catigua* A. Juss. (Meliaceae)— species not listed in the Pharmacopoeia	Interactions: front of antidepressant effects experimentally verified with mechanisms of inhibition of the reuptake of serotonin and dopamine; catuaba *Trichilia catigua* can theoretically increase the therapeutic and adverse effects of other antidepressants with the same mechanism (e.g.,: fluoxetine, duloxetine, sertraline, fluvoxamine, amineptine, bupropion, and minaprine) and can also interact with MAO, increasing the dopaminergic effects of the plant [13, 14]

Clove vine (3)	Stimulant (stalks)	*Escherichia coli*, *Enterobacter *spp., *Aspergillus flavus* (producing aflatoxin B1) (3)	(+) *Tynanthus fasciculatus* Miers, *T*.* elegans* Miers (Bignoniaceae)	Interactions: checking for the presence of coumarin in parts of the plant allows to relate theory, its potential interaction with drugs and herbal anticoagulants [15]; it may also induce seizures in epileptic patients on medication [12]. Contraindications: (*T*. *fasciculatus*) during pregnancy, lactation, in children, in epilepsy, seizures, or hyperactivity

St. John wort (3)	Depressant and Stimulant (leaves)	*Escherichia coli*, *Enterobacter *spp., *Aspergillus flavus* (producing aflatoxin B1 e B2) (3)	(−) *Hypericum perforatum* L. (Hypericaceae)	**

*Ginkgo * (4)	Stimulant (leaves)	*Enterobacter *spp., (2)	(+) *Ginkgo biloba* L. (Ginkgoaceae) (3 lots)	Adverse events: dermatitis is likely to occur following contact with the plant. It can be irritating to mucous membranes and, if ingested or placed in the eye, it might cause periorbital edema, cheilitis, eye irritation, stomatitis, and rectal irritation. In sensitized patients, it can produce pruritus ani. Nausea and vomiting may occur after ingesting the leaf extract or the seeds. Contraindications: hypersensitivity to ginkgo; concomitant use of aspirin or other antiplatelet medication. Interactions: drugs such as anticonvulsants, anticoagulants, low molecular weight heparins, selective serotonin reuptake inhibitors, monoamine oxidase inhibitors, and thiazide diuretics. Pregnancy: pregnancy risk category C; not recommended during lactation [10]

Ginseng (4)	Stimulant (roots)	*Escherichia coli*, *Enterobacter *spp., *Aspergillus flavus* (4)	(−) *Panax ginseng* C.A. Mey (Araliaceae) corresponds to the to the roots of *Pfaffia glomerata*, called “Brazilian ginseng” or “suma”	For the roots of *Pfaffia glomerata* there is report of cases of changes in praxis of healthy elderly volunteers and an increase in sleep [16]

Guarana (4)	Stimulant (seeds)	*Enterobacter *spp., *Aspergillus flavus* (4)	(+) *Paullinia cupana* Kunth. (Sapindaceae) (4 lots)	Contraindications: hypersensitivity to guarana, arrhythmia, pregnancy, breastfeeding, and severe signs of toxicity have not been reported but the usual cautions regarding caffeine apply; guarana should not be used or used with caution in patients with cardiovascular disease, chronic headache, diabetes, gastric ulcer, and in those taking theophylline, not recommended for excessive long-term use. Pregnancy: do not use during pregnancy; do not use during lactation. Interactions: drugs such as clonazepam, diazepam, alendronate, cimetidine, theophylline, and pantoprazole. Adverse effects: agitation, insomnia, nervousness, restlessness, gastrointestinal irritation, serious: arrhythmias (at high doses), palpitations (at high doses), tachycardia (at high doses), and excessive central nervous system stimulation [10, 17]

Jatob$\overset{´}{\text{a}}$ (3)	Stimulant (barks)	*Enterobacter *spp., *Aspergillus flavus* (2)	(−) *Hymenaea courbaril* L. (Fabaceae)	**

Passion fruit (4)	Depressant (leaves)	*Enterobacter *spp., *Aspergillus flavus* (3)	(+) *Passiflora alata* Curtis and *Passiflora edulis* Sims (Passifloraceae) (3 lots)	Adverse effects: Information is limited concerning toxicity of *Passiflora*. The herbal extract has been used in American traditional medicine for many years and has not generally been associated with acute or chronic toxicity. However, the pharmacological profile of the extracts of this plant suggests that large doses may result in CNS depression and ventricular dysrhythmias. Trace amounts of cyanogenic glycosides have been found in *Passiflora* species, but cyanide poisoning due to these glycosides has not been reported nor is it expected. Interactions: barbiturates, benzodiazepines; it is theoretically possible that excessive *Passiflora* doses may potentiate the effects of monoamine oxidase inhibitor drugs or might cause MAOI-type interactions with other drugs or food but these effects have not yet been documented in clinical studies or reports. Contraindications: not to be used during pregnancy. Range of toxicity: acute toxicity of *Passiflora incarnata* appears to be minimal. One human case of toxicity occurred possibly due to an inherited enzyme metabolizing defect resulting in toxic levels of the herbal, with resultant nausea, vomiting, CNS depression, prolonged QTc interval, and ventricular tachycardia [10, 11]

Marapuama (4)	Stimulant (stem barks)	*Enterobacter *spp., *Escherichia coli*, *Aspergillus flavus* (producing aflatoxin B1) (4)	(+) *Ptychopetalum olacoides* Benth. (Olacaceae)	Interactions: although there is little information available, it was found that crude ethanolic extract of marapuama increases the amphetamine-induced toxicity in animal models (amphetamine stereotypy test, lethality of yohimbine and by reversal of reserpine ptosis). There was occurrence of convulsions, cyanosis, and increased mortality of animals used in testing [18]. Such effects contraindicate the combination of extracts of marapuama with amphetamines, as used to be common in weight loss formulas for some years in Brazil [19]

Lemon balm (2)	Depressant (leaves)	Enterobacter* *spp., (1)	(−) *Melissa officinalis* L. (Lamiaceae)	**

Mulungu (4)	Depressant (barks)	*Enterobacter *spp., *Aspergillus flavus* (4)	(−) *Erythrina mulungu* Mart. ex Benth. (Fabaceae)	**

Node of dog (4)	Stimulant (roots)	*Escherichia coli*, *Enterobacter *spp., (3)	(−) *Heteropterys aphrodisiaca* O. Mach. (Malpighiaceae). The lots match to *Vernonia* cognata Less. (Asteraceae) rhizome	Little information is available for both *H*. *aphrodisiaca* as for *V*.* cognata*, allowing consideration of risks and adverse effects.

Lime flower (2)	Depressant (leaves)	*Enterobacter *spp., *Aspergillus flavus* (producing aflatoxin B1) (2)	(+) *Tilia cordata* Mill. and *Tilia platyphyllos* Scop. (Malvaceae)	Contraindications: it topically can cause hives; frequent use of flowers in the form of tea is associated with rare cardiac damage and should be used with caution in patients with heart problems. A case of a pollinic patient sensitized to lime flower pollen (*T*. *cordata*) was reported. Collateral effects: it can cause nausea and vomiting in sensitive people [12, 20, 21]. Interactions: for its mild anxiolytic effect it may theoretically show synergy with other drugs and herbal anxiolytic [22]

Valerian (2)	Depressant (roots)	*Escherichia coli*, *Enterobacter* spp., *Aspergillus flavus* (1)	(+) *Valeriana officinalis* L. (Caprifoliaceae)	Adverse effect: chronic ingestion of valerian has been associated with headache, insomnia, agitation, and hepatotoxicity. Cardiac complications and delirium have been reported following abrupt withdrawal. Symptoms of overdose have included fatigue, lightheadedness, abdominal pain, tremor, hypotension, and mydriasis. Interactions: barbiturates, benzodiazepines, ethanol, hepatotoxic agents, loperamide, and opioid analgesics [10]

(+) Plants that have their authenticity confirmed by the Pharmacopoeia and (−) plants that had no confirmation of their authenticity.

**Data on the adverse effects were not collected due to the absence of their botanical identification.
